# The Latest Criticism

**Published:** 1892-08-06

**Authors:** 


### Passing Topics.

### THE LATEST CRITICISM.

It may be said of more than one distinguished author that he drew his inspiration from the skies, and we may regard it as a genuine if unconscious tribute to our hospitals that Mr. C. S. Loch, the capable and devout exponent of all that is severe and repressive in charitable ethics is yet so far appreciative of the beneficent influences of medical charity, that when with a curious disregard of his prosaic surroundings he ventures upon metaphor, in his article contributed to the anent number of the *Nineteenth Century*, he can think of no more appropriate illustration of the hospitals than to liken them to a galaxy of stars upon the firmament.

It is true Mr. Loch reckons the hospitals as very minor stars. To him they cluster like the Pleiades, a courteous way no doubt of intimating that individually they are undistinguishable and anonymous, and that collectively they possess only trifling illuminative power. But the managers must be thankful for minute mercies, and after all the criticism as we understand it, is not that the hospitals are too small, but that they are too numerous and too lavish of their powers.

This complaint is not new, and in its reference to some part of the work of the outpatients' departments it is no doubt as true as it is venerable. No sensible man will seek to defend the plan, too generally favoured as we fear, of allowing persons to take advantage of charitable relief without adequate proof that they are eligible by reason of his poverty. On the other hand it is only fair and reasonable criticism to question the propriety of dispensing drugs regardless of the fact that the patient is so poor as to have no means of obtaining necessary food. It may be readily admitted that an alliance of medical with general charity of a nature calculated to obviate these defects would be beneficial, that is if this admission be not taken to mean that the hospitals are to be allowed to minister only to those whose merits an independent body—the C.O.S. for example—should vouch for. Hospital authorities must remain masters in their own houses, and if for no other reason because to remit cases for inquiry and report would be productive of delay and uncertainty, unjustifiable and barbarous in dealing with sick people, and utterly opposed to the instincts both of medical science and medical benevolence.

Seeing the indisposition there is in the hospital world to accept the suggestion of a central board, even though it is true of the hospitals themselves and is granted no powers of control, we may be quite sure that external interference would never be submitted to. Mr. Loch's regret that "the Select Committee have not proposed through representatives to connect the Board with general charity as in the case of the Paris Board of Supervision" has an ominous sound, and no one so well informed as Mr. Loch can fail to understand that the conditions are essentially different in the one case and the other. We can endorse every line of his concluding sentence in favour of common help and devotion, and not less warmly because we have already expressed the opinion that it is desirable both in the interests of the hospitals and for the greater perfection of their work that they should agree to act together, but we are not sure that the hospitals will be more disposed to take kindly to this counsel because it is supported by a body, the sincerity and disinterestedness of whose attachment have never been made quite plain.

